# A conditional judgment procedure for probing evaluative conditioning effects in the absence of feelings of remembering

**DOI:** 10.3758/s13428-023-02081-w

**Published:** 2023-09-20

**Authors:** Christoph Stahl, Jérémy Bena, Frederik Aust, Adrien Mierop, Olivier Corneille

**Affiliations:** 1 University of Cologne, Cologne, Germany; 2 UCLouvain, Louvain-la-Neuve, Belgium

**Keywords:** Memory, Attitudes, Evaluative conditioning

## Abstract

Attitude research has capitalized on evaluative conditioning procedures to gain insight into how evaluations are formed and may be changed. In evaluative conditioning, a conditioned stimulus (CS; e.g., an unfamiliar soda brand) is paired with an unconditioned stimulus (US) of affective value (e.g., a pleasant picture). Following this pairing, a change in CS liking may be observed (e.g., the soda brand is liked better). A question with far-reaching theoretical and practical implications is whether the change in CS liking is found when participants feel they do not remember the CS–US pairings at the time an evaluation is produced about the CS. Here, we introduce a new conditional judgment procedure—the two-button-sets (TBS) task—for probing evaluative conditioning effects without feelings of remembering about the valence of the US paired with the CS. In three experiments, the TBS is (1) is successfully validated; it is also used to (2) provide preliminary information on the feeling of remembering question, and (3) to examine an affect-consistent bias in memory judgments for CS–US pairings. Results do not support evaluative effects in the absence of feelings of remembering, and they oppose the view that affect-consistent bias is limited to memory uncertainty. We discuss these findings in light of previous evidence and of dual-learning models of attitudes. We also discuss limitations and research avenues related to the new procedure.

Over the past two decades, a wealth of evaluative conditioning studies have advanced our understanding of how evaluations may be formed and changed. In evaluative conditioning studies, a change in the evaluation of a conditioned stimulus (CS) may be observed following its pairing with an affective stimulus (US). In this literature, researchers have long been interested in whether evaluative conditioning (EC) effects are found in the absence of awareness of the CS–US pairings. To address this question, *experimental studies* have suppressed the encoding of the CS–US pairs at the learning stage, and examined what remained of the EC effect under such testing conditions. In general, these studies found no support for the unconscious EC hypothesis. For instance, when stimulus strength is low or when a secondary task prevents the successful encoding of the CS–US pairings, an evaluative conditioning effect is unlikely to be elicited (for a recent review, see Corneille & Stahl, 2019). In contrast, *correlational studies* have classically used memory measures as a proxy for the (successful or unsuccessful) encoding of the CS–US pairs. This second line of research brought mixed support to the possibility of EC without CS–US pairing awareness (see below). In the last decade, however, it became increasingly apparent that that this second strand of – correlational – research examined relations between memory and evaluations, rather than the role of encoding performance in attitude acquisition (for instance, see Gawronski & Walther, 2012). As a result, evaluative conditioning research became increasingly interested in linking the question of evaluative formation and change to memory models (e.g., Gast, 2018: Stahl & Aust, 2018).

Furthermore, there are reasons to believe (as we will argue below) that this research has examined EC in the absence of subjective feelings of remembering rather than memory performance (see also Waroquier et al., 2020). The question of whether EC can be found without participants’ feeling of remembering US valence is important for both theoretical research (e.g., for models of the role of memory processes in EC; for theories of implicit learning and consciousness) and applied research (e.g., on the controllability of the effects of persuasive influences on attitudes and behaviors).

Below, we first provide an overview and discussion of how recent research has addressed EC effects as a function of memory performance, and what conclusions were derived from two main procedures: the per-item and process dissociation procedures. In a second step, we introduce a novel procedure that combines the advantages of the two prior ones. In the empirical section, we validate the new procedure and use it to provide firmer conclusions about the contingency of EC effects upon feelings of remembering US valence. In doing so, we also shed additional light on affect-as-information processes. In the General discussion, we summarize the main insights of the new procedure introduced here, and we explain how it can be used in future research (i.e., attitude research and beyond).

## A short overview of research on the memory question

Recent research developed and relied on two categories of procedures to examine associations between EC effects and memory. A first line of research relied on per-item procedures. A second line of research made use of a process dissociation procedure.

The per-item rationale consisted of examining if evaluative conditioning effects are found for CSs that participants prove unable to pair with the identity or the valence of their associated US in a separate memory task. As also true for any awareness measure, assessments of CS–US pairing memory are most informative and relevant if they meet certain criteria (Shanks & St. John, 1994). Among those criteria is the information criterion: the requirement that assessments tap memory for those bits of information that are critical in producing the effect in question. The information criterion is ideally met (1) by assessments made at the level of the specific CS item (as opposed to aggregating across items and classifying participants as aware or unaware), and (2) by assessing memory for US valence (rather than US identity). When relying on per-item procedures, researchers generally found no EC effect for CSs associated with incorrect memory reports in a separate memory task (Pleyers et al., 2007; Stahl et al., 2009). In addition, memory for US valence rather than memory for US identity proved to be critical for obtaining evaluative conditioning effects (Stahl et al., 2009). While per-item procedures meet the information criterion, they however fail to meet the immediacy criterion: the requirement that awareness should be assessed at the time when the relevant cognitive process occurs (i.e., the one participants’ awareness is about), not later (Mitchell et al., 2009). In per-item procedures, evaluations of all stimuli are typically elicited in a rating task, whereas memory is assessed in a different, independent task. Thus, in per-item procedures, the memory assessment is temporally separated from CS evaluations.

In contrast to per-item procedures, the Process Dissociation procedure for studying EC in the absence of memory (Hütter et al., 2012) aimed at separating, at the aggregate level and within a single task, participants’ memory for US valence from their attitude towards the CS in the absence of such explicit memory. In the Process Dissociation procedure, participants indicate for each CS whether the CS was paired with pleasant or unpleasant USs (by selecting either a “pleasant” or “unpleasant” response). Alternatively, if participants cannot remember the valence of the US, they have to indicate whether they find the CS pleasant or unpleasant (using the same set of “pleasant” or “unpleasant” responses). Participants thus perform a two-stage decision within a single task: First, they decide whether they remember US valence or not. The outcome of this decision determines how to proceed in the second stage—report the memory judgment from the first stage (if they remember US valence) or perform and report an attitude judgment (if they don’t remember US valence). Using multinomial processing modelling, these procedures found a small but significantly above-zero estimate for a parameter that was interpreted as indicative of US-consistent evaluations without conscious CS–US pairing memory. As classically achieved in Process Dissociation procedures (Jacoby, 1991; for review see Yonelinas & Jacoby, 2012), this was done by comparing response frequencies in conditions where attitudes towards the CSs and memory for their paired US valence operated synergistically (inclusion condition) versus antagonistically (exclusion condition).

Both per-item and process dissociation procedures have delivered valuable insights in evaluative learning research. These procedures, however, come with some limitations. One important limitation applying to per-item procedures is that they involve two separate measurements of CS evaluations and US memory. These multiple assessments imply additional measurement error. This is less of a problem in the process dissociation procedure as it involves a unique measurement for each CS. However, contrary to per-item procedures, data collected in process dissociation procedures are interpretable only at the group aggregate level (i.e., parameter estimates computed across inclusion and exclusion groups, based on data aggregated across items and persons). Thus, these procedures do not conform to the information criterion, as they fail to deliver information about memory judgments or evaluations by a specific person about a specific CS. This restriction furthermore prevents addressing additional research questions by relating memory states to evaluations at the item level (see also the General discussion).

Here, we introduce a new procedure that makes the best of the two previous approaches while overcoming some of their limitations. Before doing so, however, it is important to discuss how these and complementary approaches have dealt with a decision heuristic that may contaminate memory assessments: the affect-as-information heuristic.

### The affect-as-information bias and how it has been dealt with

When participants lack memory about the valence of the USs paired with a CS, but are nevertheless asked to report it, they may respond on the basis of their feelings toward the CS (e.g., “the CS feels pleasant, so it must have been paired with positive USs”). This response pattern may then be mistakenly interpreted as indicating that an EC effect was accompanied by US valence memory. As a result, researchers are at risk of rejecting from the “US-unaware” category those CSs for which no conscious US valence memory existed but for which “false US valence memory” was rather inferred from CS evaluation. Hence, affect-as-information can make per-item analyses too conservative a test for the possibility of evaluative conditioning effects in the absence of US valence memory.

As a side comment, the affect-as-information heuristic may explain reversed evaluative conditioning effects sometimes found for CSs associated with incorrect valence-memory responses (i.e., evaluative shifts and memory judgments inconsistent with actual US valence). The effect is assumed to be based on pre-experimental CS attitudes that are opposite to the valence of the US. If US valence memory is absent for those CSs, then participants may use their pre-existing attitude to inform both their US memory judgment—leading to an incorrect valence-memory response—and their CS evaluations—leading to an evaluative effect opposite to the US’s valence (i.e., a reversed EC effect). Such reversed EC effects for CS with incorrect memory responses were found in Stahl et al. (2009, Experiment 3; meta-analysis of Experiments 2–4).

Because of this contamination issue, studies have attempted to minimize, control, or assess the role of affect-as-information in the measurement of US valence memory. In studies relying on the per-item procedure, the contribution of affect-as-information was dealt with by introducing an “I don’t remember” response option in the memory task, thereby allowing participants to indicate their lack of memory (instead of forcing them to guess). In doing so, researchers avoided pressuring participants into reporting a CS–US pairing memory they did not feel confident about. In Process Dissociation studies, the issue is similarly avoided as participants are not forced to guess the US valence: if participants think they do not remember the US valence, they are instructed to report their attitude, not their memory response.

The above discussion highlights that, to avoid forcing participants to guess, both procedures have asked participants to report their memory of US valence only if they felt they could retrieve it. Hence, both per-item and process dissociation methods eventually captured participants’ subjective feelings of remembering (or not) the US valence. This is an important qualification, considering that both procedures initially considered memory measures as proxies for the successful encoding of the CS–US pairs at the learning stage (rather than as indexes of feelings of remembering US valence at the judgment stage). This interpretational updating is perhaps best illustrated in a recent study by Hütter & De Houwer (2017) showing that estimates of the memory parameter are affected by criterion manipulations. As these authors acknowledged: “MPT estimates could depend on the subjective criteria that participants use to decide whether they remember or do not remember the CS–US pairings.” (p. 51).

To sum up, recent developments in EC research acknowledge that memory studies (1) ultimately dealt with associations between memory and EC effects (rather than with the role of encoding performance in attitude acquisition), and (2) likely captured (although unintendingly) subjective feelings of remembering in addition to objective memory accuracy. In other words, these methods have informed us about EC effects (not in the absence of memory but) in the absence of feelings of remembering. The method introduced in the remaining of this introduction aims at further improving the assessment of EC effects in the absence of feelings of remembering.

### Introducing the Two-Button-Sets (TBS) task

**Fig. 1:**
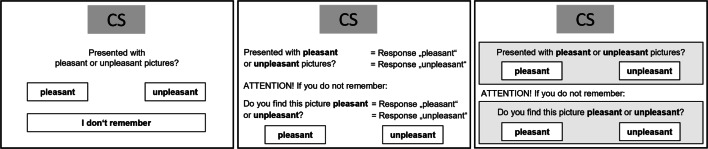
Screen displays of the per-item valence-memory task (*left*), the process-dissociation task (*center*, inclusion condition), and the TBS task (*right*)

We introduce here a new conditional judgment procedure: the two-button-sets (or TBS) task for estimating EC effects in the absence of feelings of remembering the US valence paired with the CS. The TBS display is illustrated in Fig. 1 (right). For reference, the figure also illustrates the per-item and process dissociation task displays (left and center). Contrary to Hütter et al., (2012)’s process dissociation procedure, the TBS introduces two sets of “pleasant”/“unpleasant” response buttons. The first set is designated for memory judgments. The second set is designated for attitude judgments. On a given trial, only one judgment is reported, conditional upon the feeling of remembering: Whenever participants felt that they remember US valence, they are instructed to use the Memory responses set (in the upper half of the screen) to report the outcome of their memory judgment. Alternatively, if they felt that they do not remember, participants are instructed to use the Attitude responses set (in the lower half of the screen) to report the outcome of their attitude judgment.

**Fig. 2:**
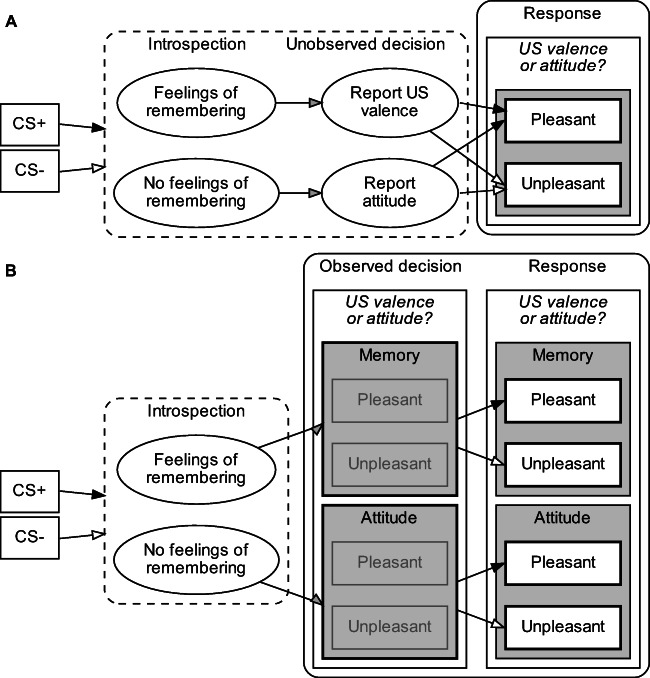
Schematic comparison of the two-stage tasks in the process-dissociation (**A**) and TBS tasks (**B**). In both the process-dissociation and TBS tasks, participants first introspect whether they remember US valence or not. If they do remember US valence, they report their US-valence memory judgment; if they do not remember US valence, they report an attitude judgment. While the decision to report either a memory or attitude judgment remains unobserved in the process-dissociation task (A), it is observed as the choice between Memory and Attitude button sets in the TBS task (B). Panels with *dashed lines* represent unobservable latent states; those with *solid lines* observed task components. *Bold rectangles* represent observable response options at each task stage. *Black and white arrows* represent paths for positively and negatively paired CSs, respectively; *grey arrows* represent common paths

The distinctive feature of the TBS is that it renders metacognitive decisions (i.e., whether participants feel they remember the US valence) observable at the item-within-person level (see Fig. 2). This has three important advantages. First, because the remember decision is unveiled, it is no longer necessary to infer it indirectly from parameter estimates of a multinomial processing tree model, as is the case in the process dissociation approach. In turn, the between-subject instruction manipulation can be omitted so that a single group of participants is sufficient. This makes the new procedure more efficient and flexible. For example, it can be used in within-participant designs that allow for more stringent experimental control as well as higher statistical power. Second, contrary to the per-item task but similar to the original process dissociation procedure, the immediacy criterion is met and the error measurement issue originating from multiple assessments of evaluation and memory is reduced. This is because a single measurement (either for US memory or for CS evaluation) is involved for each individual CS.^1^ Third, the CS-level information enables finer-grained analyses and more rigorous tests; most importantly, EC effects can be analyzed at the item-within-person instead of the group level; hence, the TBS respects the information criterion.

To sum up, the TBS combines the advantages and reduces the limitations of the previous per-item and process-dissociation procedures by introducing slight but significant departures from these two procedures. The TBS is highly similar to the per-item task: It can be seen as expanding the “I don’t remember” response into a binary CS evaluation. The TBS is even more similar to the original process dissociation task: Here, participants’ two-stage task remains the same, except that the TBS is augmented by a second set of “pleasant”/“unpleasant” buttons so that a dedicated set of buttons is used for both the presence and the absence of feelings of remembering. This adaptation of the original process dissociation task allows analyses at the item-within-person level: Beyond procedural simplification (i.e., removing the need for a between-subjects manipulation of instruction), the TBS also simplifies data-analytical assumptions (i.e., it is no longer necessary to assume identical processes in the inclusion and exclusion conditions, nor independency across participants). Instead of using a MPT model as that proposed by Hütter et al., (2012) to produce what are hoped to be process-pure estimates of the cognitive processes of interest (in particular, EC in the absence of memory) at the group level, the TBS obtains per-item responses that are not claimed to be process pure but can be easily analyzed together with the results of other measures to investigate and scrutinize their validity. The TBS simplifies the original process dissociation procedure and provides important insights into metacognitive processes involved in participants’ decisions at the item level (i.e., feelings of remembering the valence of the US paired with a given CS).

These assets are helpful for examining the role of feelings of remembering in EC and the affect-as-information question (see also below). As we will discuss in the general discussion, they are also helpful for addressing complementary questions. Observable decisions at the item level also allows assessing the accuracy of participants’ feelings of remembering (e.g., the proportion of “remember” reports reflecting accurate US memory, and the proportion of “I don’t remember” cases in which correct US memory is nevertheless present). TBS data can be fruitfully related to the outcomes of other measures obtained from the same participant, for instance, evaluative (e.g., ratings) or (metacognitive) memory measures (e.g., Remember-Know-Guess or confidence judgments). Relating item-level responses to other measures allows investigating a wider range of research questions regarding memory-evaluation relations.

We now turn to the empirical section, where we validate the TBS and use it to address our current feelings-of-remembering and affect-as-information questions.

## Overview of present studies

To empirically support the new TBS task in a manner comparable to that of the established tasks, we ran two validation studies. The studies varied whether a CS was paired with a US or not, hence manipulating US memory. They also varied whether a CS was neutral or valenced, thereby manipulating CS attitudes. In addition to this validation part, we used the TBS task to provide preliminary information on whether EC can be observed in the absence of feelings of remembering US Valence.

We also investigated whether attitudes contaminate memory measures (i.e., affect-as-information bias) in ways beyond those discussed above. Bar-Anan and colleagues (Bar-Anan & Amzaleg-David, 2014; Bar-Anan et al., 2010) manipulated attitudes experimentally before assessing how they affected US valence memories. Participants’ memory reports were biased toward their evaluation of the CS, which is consistent with the use of an affect-as-information heuristic. However, the bias was found to be absent from guessing and vague-memory cases; contrary to the authors’ expectations, it was restricted to cases where participants claimed to have clear memory of a pairing. The authors concluded that “evaluation influenced memory judgement in a way that participants experienced as clear memory, although it was only a reconstructed memory judgment based on evaluative information rather than true memory traces of past events.” (p.1042). These findings importantly suggest that participants may rely on affect-as-information decision making, but not necessarily to overcome vague memories. In other words, they suggest that attitudes may bias memory measures even—or especially—when participants are confident of their memories and experience them as clear recollections. Yet, the findings reported by Bar-Anan and colleagues were based on old/new recognition judgments of specific CS–US pairs instead of valence-memory judgments, and so it is unclear how they affect valence-memory measures. Using the new procedure proposed above, we will be able to provide answers to this complementary question.

An additional goal of the present line of research was to compare the TBS with the process-dissociation procedures with a focus on testing the process-dissociation approach’s underlying assumptions. To mirror the process-dissociation procedure, in addition to the standard TBS instruction condition we also ran a group that worked on a TBS variant of the Exclusion instruction, and we collected additional memory measures at the end of the study. For the sake of conciseness and because these complementary analyses were not directly relevant to our present validation goal, the present article does however not address this issue, and the data from that condition were not included in the analyses reported below. Hence, we omitted the TBS Exclusion condition and these additional memory measures from the empirical section (the data from these conditions and measures will be reported elsewhere and made available in the OSF repository).

To sum up, the present study is primarily aimed at introducing the TBS and gathering empirical support for its validity for investigating how remembering US valence relates to EC effects. In doing so, this research provides preliminary information about whether EC is found without feelings of remembering US valence, and information relevant to the attitude-as-information bias. These latter—more theoretical—goals, however, should be considered more secondary in the context of the present study.

## Experiment 1

**Fig. 3:**
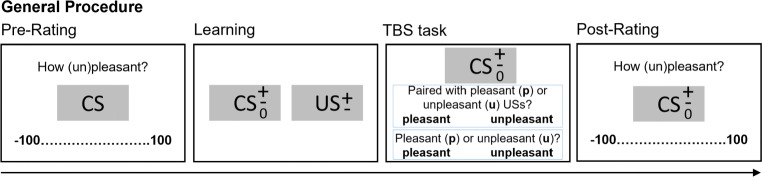
Illustration of general procedure. CS items were pre-rated and then assigned to within-subject conditions. After the learning phase, the TBS task was administered, followed by post-learning ratings. Exp.3 deviated from this procedure in that one of three valence-memory tasks (TBS, VMA, or 3ACE) was administered, and the order of valence-memory task and post-study evaluative ratings was counterbalanced

Experiment 1 probes the validity of the TBS task by manipulating both CS–US pairing memory and CS attitudes (within subjects) to test whether the manipulations selectively affect the Memory-set and Attitude-set responses. CS stimuli were selected individually on the basis of a given participant’s pre-study ratings. To manipulate pairing memory, only the neutrally pre-rated CSs (but not the valent foils) were paired with USs during learning. This should affect the frequency of Memory-set responses for the neutral CSs (compared to the valent foils). To manipulate CS attitudes, the most valent (positive and negative) foil stimuli were compared (that were not paired with USs during learning): For these stimuli, Attitude-set responses should reflect pre-rated attitudes. Figure 3 gives an overview of the experimental procedure.

### Method

For the present studies, we report how we determined our sample sizes, all data exclusions (if any), all manipulations (except the inclusion/exclusion factor, as explained above), and all measures (except the memory measures that were collected after the TBS and post-rating blocks, as explained above). The study was registered prior to data collection and analysis (see https://osf.io/rkb3w; deviating from the preregistration, the present article does not report the analyses involving the inclusion/exclusion factor). Materials, data, and analysis scripts are available at https://osf.io/ygcz4.

#### Participants and design

The study realizes a 2 x 2 design with repeated measures: Valence (positive vs. negative) x Pairing (critical/paired vs. control/unpaired). We collected a sample of *N* = 60 participants (students at UCLouvain), as this is approximately 2x the sample size recruited by Hütter et al., (2012) (as recommended by the rule of thumb for replications by Simonsohn, 2015). We had to exclude the data from 1 participant who was accidentally run in both the inclusion and exclusion condition, and from 17 participants due to errors on instruction checks; the remaining sample consisted of *N* = 42 participants.

#### Materials and procedure

We used the same materials as Hütter et al. (2012): A set of photographic images of faces served as CSs; a set of IAPS images served as USs. The face CSs were pre-rated by participants by positioning a cursor on a slider ranging from ‘very unpleasant’ (-100) to ‘very pleasant’ (+ 100). Of these faces, the 24 most neutrally pre-rated ones served as critical CSs (i.e., 12 paired with positive USs, 12 with negative USs). The 16 most extremely pre-rated faces (8 of each valence) were not presented during the learning phase but served as valent control stimuli. During learning, the 24 critical CSs were repeatedly paired with USs (i.e., each CS was presented once with one of eight USs of the same valence). Dependent measures were collected for all 40 CS stimuli (i.e., the two sets of 24 critical/paired and 16 control/nonpaired face photographs).

The procedure closely followed that of the Hütter et al. (2012) studies. First, participants pre-rated all 120 face stimuli. In the subsequent learning phase, CS and US images were presented side by side on the computer screen (positions were randomly selected for each trial). After learning, memory and attitude were assessed for all 40 CSs. The TBS task was implemented first. After instructions were given, instruction understanding was tested: Participants were presented with descriptions of the possible combinations of memory and attitude states (one for each possible case), and were asked to select the corresponding response according to instructions. TBS was followed by post-learning evaluative ratings.

#### Data analysis

As a manipulation check, the pre-post change in CS evaluations was computed (i.e., post-learning evaluation minus pre-learning evaluation) and submitted to a repeated-measures ANOVA to probe for US valence effects. The TBS responses (i.e., choice of Memory vs. Attitude response-button sets; pleasant vs. unpleasant response) were analyzed using logistic regression analyses, with by-person random intercepts and slopes for the experimental factors. In Experiment 1, random effects for interactions were also added; this was not possible in Experiment 2 because of the more complex design, which yielded a more complex model that was not supported given the relatively limited data. Omitting the interaction terms from the random-effects structure of the model did not reduce the model’s goodness of fit, *χ*^2^(30) = 9.69, *p* = .99.^2^

### Results

We first report results of a manipulation check (i.e., whether our study produced EC effects). The responses on the novel TBS task are analyzed next, first assessing its validity (i.e., whether the memory and attitude manipulations were adequately reflected in the responses), and then testing for EC without feelings of remembering US valence as well as for attitude-as-information bias. In Appendix A, we report additional results on the proportions of Memory and Attitude-set judgments and on valence memory accuracy in each response set.

#### Evaluative conditioning

**Fig. 4:**
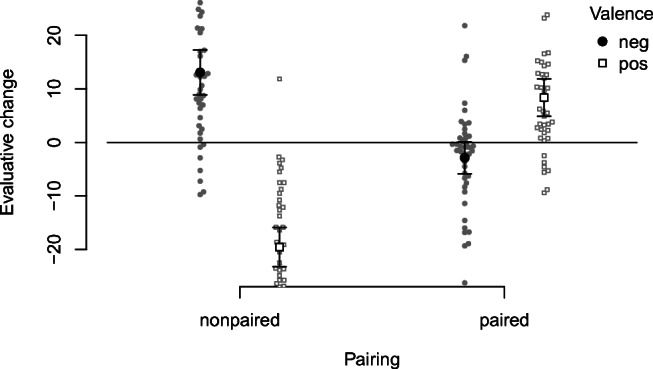
EC effects (pre-post evaluative changes) as a function of Valence and Pairing

Evaluative change was computed as post-learning evaluation minus pre-learning evaluation and submitted to a 2 (Valence) x 2 (Pairing) repeated-measures ANOVA. Figure 4 shows the results.

EC was affected by Valence, *F*(1,41) = 34.28, *p* < .001, *η̂G*2 = .180, 90% CI [.037,.351], Pairing, *F*(1,41) = 17.26, *p* < .001, *η̂G*2 = .064, 90% CI [.000,.214], as well as, most strongly, their interaction, *F*(1,41) = 100.87, *p* < .001, *η̂G*2 = .480, 90% CI [.294,.615]. For nonpaired CSs, pre-post changes reflected the statistical artifact of regression to the mean: The CSs with most extremely positive pre-ratings became less positive, and the CSs with most extremely negative pre-ratings became less negative. For paired CSs, pre-post evaluative changes reflected US valence (i.e., an EC effect): Those paired with positive CSs became more positive, while those paired with negative USs became more negative. Interestingly, there was an asymmetry, with the positive larger than the negative shift.

#### Validation of the two-button-sets procedure

To show that the modified task adequately assesses metamemory judgments of CS–US pairings, and attitudes for cases in which participants do not remember CS–US pairings, we analyzed whether (1) pairing affects participants’ Memory-set reports (which we expected to differ between paired versus nonpaired control CSs); (2) those memory reports are largely accurate; and (3) participants’ pre-rated attitudes are consistently reflected in the Attitude button responses.

Table 1 shows response frequencies on the TBS task. The first columns show that participants’ metamemory judgments clearly distinguished quite well between paired and nonpaired stimuli, *χ*^2^(1) = 574.85, *p* = .001. 3

**Table 1:** Response frequencies (proportions) on the TBS task, separated by pairing status and US valence (Experiment 1)

Set	CS	Foil	Response	CS +	CS−	Foil +	Foil−
Memory	652	39	+	270 (.41)	58 (.09)	24 (.62)	2 (.05)
	(.65)	(.06)	-	66 (.10)	258 (.40)	4 (.10)	9 (.23)
Attitude	356	633	+	86 (.24)	89 (.25)	295 (.47)	11 (.02)
	(.35)	(.94)	-	82 (.23)	99 (.28)	13 (.02)	314 (.50)

Next, we analyzed the frequencies of “pleasant”/“unpleasant” responses. They varied as a function of Valence, *β̂* = 1.95, 95% CI [1.50,2.39], *z* = 8.56, *p* < .001, and this effect was modulated by a two-way interaction with Pairing, *β̂* = 1.14, 95% CI [0.70,1.57], *z* = 5.14, *p* < .001, as well as its three-way interaction with Response Set (Memory vs. Attitude), *β̂* = − 0.88, 95% CI [− 1.19,− 0.57], *z* = − 5.57, *p* < .001. Separate analyses for paired and nonpaired stimuli showed, for both item types, effects of Valence (paired: *β̂* = 0.80, 95% CI [0.60,1.00], *z* = 7.76, *p* < .001; nonpaired: *β̂* = 3.17, 95% CI [2.20,4.15], *z* = 6.38, *p* < .001). Importantly, they also showed interactions of Valence with Response-Set (paired: *β̂* = 0.71, 95% CI [0.56,0.87], *z* = 9.16, *p* < .001; nonpaired: *β̂* = − 0.99, 95% CI [− 1.59,− 0.38], *z* = − 3.19, *p* = .001), indicating that Valence differentially affects Memory and Attitude responses. We therefore investigated the effect of Valence separately for each of the four subsets of the Pairing (paired CS vs. nonpaired foil) x Response Set (Memory vs. Attitude).

The corresponding aggregate counts are given in the four 2-by-2 quadrants of Table 1 (one for each combination of the Pairing and Mem/Att variables). The top left quadrant (i.e., Memory judgments for paired stimuli) is relevant for evaluating accuracy of Memory responses. It shows frequencies (and proportions, in parentheses) of ‘pleasant’ (Val:+) and ‘unpleasant’ (Val:-) responses for CSs that were paired either with positive (CS + ) or negative (CS−) USs. Memory judgments for paired stimuli were affected by (i.e., quite accurately reflected) actual US valence, *β̂* = 1.64, 95% CI [1.30,1.98], *z* = 9.53, *p* < .001. When participants experienced remembering US valence for a paired stimulus, they were accurate in 80% of cases.

The bottom right quadrant (i.e., Attitude judgments for nonpaired stimuli) informs us about the task’s ability to reflect pre-experimental attitude. Nonpaired stimuli (i.e., those not presented together with USs) were selected to be either clearly positive or negative for a given participant. Attitude judgments for nonpaired valent stimuli were strongly influenced by (i.e., quite accurately reflected) participants’ pre-study attitudes towards these stimuli, *β̂* = 4.98, 95% CI [2.90,7.06], *z* = 4.70, *p* < .001. Participants consistently reported the attitude corresponding to their pre-study ratings in 95% of cases. So far, the results show that the TBS task adequately reflected experimental manipulations: Participants’ metamemory judgments were able to separate paired from nonpaired stimuli; Memory-set judgments for paired CSs were largely accurate; and Attitude-set judgments reflected participants’ attitudes quite well.

The remaining two quadrants inform us about two relations between EC and memory: EC without feelings of remembering US valence (bottom-left quadrant), and attitude-as-information bias (top-right quadrant).

#### Is EC observed without feelings of remembering US valence?

The bottom left quadrant (Attitude-set judgments for paired stimuli) is relevant to the issue of EC in the absence of feelings of remembering: An Attitude-set judgment implies the metacognitive absence of remembering US valence. For paired CSs, the Attitude-set responses should reflect evaluations in the absence of remembering US valence. We saw above that Valence affected responses to paired CSs, and that this effect was modulated by an interaction with Response-Button set: Whereas the effect of Valence was found for Memory-set responses (see above), it was absent from Attitude-set responses, *β̂* = 0.08, 95% CI [− 0.18,0.35],*z* = 0.61, *p* = .544. Thus, as reflected in Table 1, there was no significant EC in the absence of feelings of remembering US valence.

Because failing to find a significant effect is not evidence for the absence of an effect, we additionally conducted a Bayesian analysis that allows quantifying the evidence for the null hypothesis of no effect. Specifically, for Attitude-set judgments on paired neutral CSs, we computed a Bayes factor (*BF*) on the proportions of responses in line with US Valence. We modeled the distribution of true effect sizes under the *H1* as a half-normal distribution with an *SD* of 15% (similar to Waroquier et al.,, 2020).^4^ For each *BF*, noted *BF*_*H1*:*N*(0,.15)_, we report the Robustness Region to indicate the range of *SD* s of the *H1* distribution that support the same conclusion as the reported *BF*; the *SDs* ranged from zero to the maximal value of the scale (i.e., 100%). We used conventional cut-offs to interpret *BF* s (e.g., Dienes, 2014): A *BF* above 3 yields evidence for *H1* compared with *H0*, while a *BF* below 1/3 yields evidence for *H0*. A *BF* between 1/3 and 3 would count as anecdotal evidence in either direction, and indicates inconclusive data (i.e., no evidence for either hypothesis).

The Bayes factor analyses yielded inconclusive evidence for or against above-chance responses in line with US valence (*M* = 0.53; *SD* = 0.23), *BF*_*H1*:*N*(0,0.15)_ = 0.58 (Robustness Region: 0.05, 0.25). In sum, there was no significant EC effect for Attitude-set judgments, but we cannot conclude from this finding that there is evidence for no EC in the absence of feelings of remembering US valence.

#### Is there evidence for an affect-as-information bias?

Finally, we examined whether Memory-set responses were systematically affected by participants’ attitudes towards nonpaired (but inherently valent) stimuli. The top-right quadrant of Table 1 (Memory-set judgments for nonpaired stimuli) serves as a test for attitude-consistent guessing. Most often, nonpaired stimuli were judged ‘not remembered’, and hence Attitude-set responses were given on the TBS task. Yet, in those cases that participants falsely ‘remembered’ that a US had been paired with them (i.e., used the Memory button set), they reported a US valence that tended to be consistent with pre-experimental attitudes, *β̂* = 10.16, 95% CI [2.56,17.75], *z* = 2.62, *p* = .009. (However, as indicated by the above interaction with Response Set, this effect was smaller than for the Attitude-set judgments.) In sum, participants indeed showed an attitude-as-information bias on Memory-set responses: For a stimulus falsely judged as having been paired with a US, they relied on their evaluation of that stimulus to inform their memory judgments in 73% of cases. Yet, Memory-set responses did not reflect attitudes as strongly as did Attitude-set responses. This suggests that participants may be able to influence the degree to which they allow pre-existing attitudes to inform their judgments.

Taken together, the results of the TBS task appropriately reflected the memory and attitude manipulations. Furthermore, we did not find an EC effect in the absence of feelings of remembering US valence. In addition, an effect of attitude-as-information was found in Memory-set responses for nonpaired stimuli.

### Discussion

Results of Experiment 1 largely confirmed expectations: First, the pairing manipulation was effective: a standard EC effect was found (along with a regression-to-the-mean effect for pre-experimentally valent stimuli), and the TBS task was successfully validated: The choice of Memory-set versus Attitude response-button set reflected participants’ metacognitive *remember* judgments (i.e., robustly higher rates of memory Memory-set responses for paired than non-paired stimuli); and Attitude-set responses reflected pre-learning evaluative ratings.

Second, we extended previous findings on memory-evaluation relations: In line with Stahl et al., (2009), we found no EC when participants felt that they did not remember US valence. In line with Bar-Anan et al., (2010, 2014), we found memory judgments for nonpaired stimuli to be biased by attitudes: Participants’ valence-memory responses tended to be consistent with their pre-experimental ratings towards these stimuli. This bias was obtained despite metacognitive ‘remember’ judgments, suggesting that participants were at least somewhat confident about their memories.

Note that Experiment 1 had several limitations. Perhaps most importantly, as in previous studies, pairing memory and (pre-existing) attitude were confounded: Onlyneutrally pre-rated CSs were paired with (positive or negative) USs; valent CSs were never paired. This means that we could not assess the simultaneous effects of pairing and pre-experimental attitudes on the same set of CSs. Experiment 2 addressed these limitations.

## Experiment 2

Experiment 2 examines the robustness of the findings from Experiment 1 and extended it in several ways: First, memory and attitudes were manipulated in a fully orthogonal within-subjects design by independently varying CS valence (positive, neutral, negative) and US valence (positive, non-paired, negative). Second, new US material was used, and CS Material (human faces vs. toy figures) was additionally varied between subjects (this was done for generalization purposes; we did not expect any effects). To control for carry-over effects on these measures from the TBS task, another group of participants was added that worked only on the latter two memory measures.^5^

### Method

The study was preregistered at https://osf.io/mxtwd(here we focus on validating the TBS task; the analyses addressing the other goals will be reported elsewhere). Material, data, and analysis scripts are available at https://osf.io/ygcz4.

#### Participants and design

Participants were randomly assigned to a 2 (*TBS*: present vs. absent) x 2 (*Material*: human faces vs. toy figures) between-subject design. The sample consisted of *N* = 76 students from the University of Cologne (*n*_*TBS*−*present*_ = 36,*n*_*TBS*−*absent*_ = 40).^6^ Within subjects, *US Valence* (positive, neutral/non-paired, negative) and *CS Valence* (positive, neutral, negative), were manipulated orthogonally.

#### Material

As in Experiment 1, images of faces were used as CSs (deviating from Exp.1, we selected only the first 100 from the set of 120 images). As additional CSs, we used 100 photographs of toy figures (*Gogo Crazy Bones*). As USs, we used 112 color images from the OASIS database (Kurdi, Lozano, & Banaji 2017; 56 positive and 56 negative, with comparable arousal ratings). For a given participant, the most neutrally pre-rated 24 faces or toys as well as the 18 most valent faces or toys (nine positive, nine negative) served as CSs. Of the 24 neutral CSs, 16 were paired with USs (eight positive, eight negative). Of the initially valent CSs, three from each valence were paired with USs of each valence (i.e., three initially positive as well as three initially negative CSs were paired with positive USs; the other three from each valence set were paired with negative USs). The remaining eight neutral CSs, together with three positive and three negative CSs, comprised the set of 14 non-paired control stimuli. A set of four different USs was randomly assigned to and uniquely paired with each CS; each US was paired twice with that CS. Where possible, dependent measures were collected for all 42 CSs; US identity memory was not assessed for non-paired stimuli.

#### Procedure

The procedure closely followed that of Experiment 1. Deviating from Experiment 1, evaluative ratings were collected on a 20-point (instead of 200-point) scale. Further deviating from Experiment 1, but in line with Hütter et al. (2012), we presented participants with up to three rounds of instructions and instruction-check items; participants with errors on the last instruction-check round were excluded from analyses. 7

### Results

As in Experiment 1, we first report the results of a manipulation check (i.e., the effects of the experimental valence manipulations on evaluative ratings). Next, we analyzed the responses on the TBS task. In Appendix A, we report additional results on the proportions of Memory- and Attitude-set judgments and on valence memory accuracy in each response set.

#### Evaluative conditioning

**Fig. 5:**
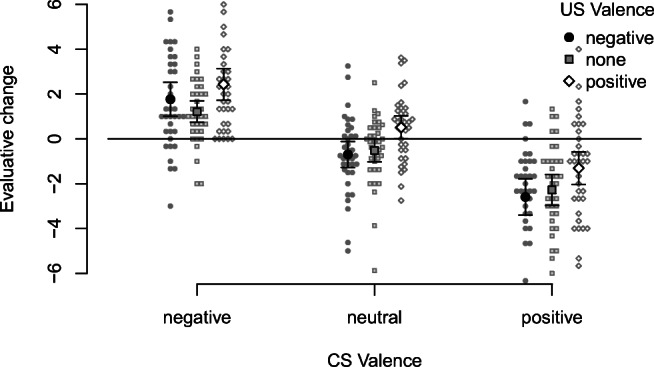
Pre-post evaluative change as a function of CS Valence and US Valence (face CS material)

**Fig. 6:**
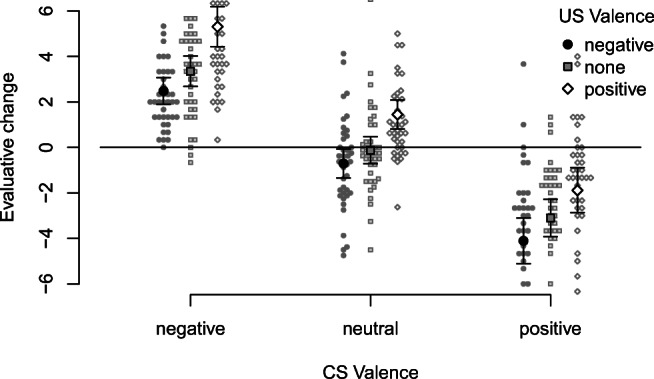
Pre-post evaluative change as a function of CS Valence and US Valence (toy CS material)

To examine EC effects, pre-post evaluative changes were entered into an ANOVA with all of the experimental factors. Figure 5 (face CSs) and Fig. 6 (toy CSs) illustrate pre-post evaluative change as a function of CS Valence and US Valence.

We found robust US valence effects, *F*(1.67,119.92) = 50.04, *p* < .001, *η̂G*2 = .106, 90% CI [.035,.185]. Compared with nonpaired stimuli, pairing with pleasant USs clearly increased liking, *F*(1,72) = 63.88, *p* < .001, *η̂G*2 = .092, 90% CI [.014,.210], whereas the reduction by unpleasant USs was considerably smaller, *F*(1,72) = 6.52, *p* = .013, *η̂G*2 = .009, 90% CI [.000,.076].

We also found a strong regression-to-the-mean artifact for pre-experimentally valent CSs (i.e., CSs selected because they were initially most negative were later evaluated more positively; and vice versa), *F*(1.49,107.13) = 232.69, *p* < .001, *η̂G*2 = .505, 90% CI [.411,.579]. Interactions with Material indicated that both effects were stronger for toy figures than faces (CS Valence: *F*(1.49,107.13) = 17.09, *p* < .001, *η̂G*2 = .070, 90% CI [.013,.139]; US Valence: *F*(1.67,119.92) = 6.86, *p* = .003, *η̂G*2 = .016, 90% CI [.000,.057]). While the increase in liking was found for both materials (faces: *F*(1,34) = 29.39, *p* < .001, *η̂G*2 = .091, 90% CI [.000,.267], toys: *F*(1,38) = 36.81, *p* < .001, *η̂G*2 = .099, 90% CI [.000,.267]), the reduction by unpleasant USs was limited to toy figures, *F*(1,38) = 14.20, *p* = .001, *η̂G*2 = .032, 90% CI [.000,.168], and absent from faces *F*(1,34) = 0.03, *p* = .862, *η̂G*2 = .000, 90% CI [.000,.000]. Interestingly, the US valence effect was not modified by CS Valence, *F*(3.62,260.80) = 0.55, *p* = .685: In contrast to the widespread notion that EC works best for neutral CSs, the EC effect was of the same magnitude for valent and neutral CSs.^8^

#### Validation of the two-button-sets procedure

The responses in the TBS task are given descriptively and in a condensed form in Table 2, which is structured as in Exp. 1 for ease of reference by pairing status and valence (positive vs. negative; neutral foils are not shown, and data are collapsed across all other factors).

**Table 2:** Response frequencies (proportions) on the TBS task, separated by pairing status and valence but collapsed across all other factors (Experiment 2)

Set	CS	Foil	Response	CS +	CS−	Foil +	Foil−
Memory	600	77	+	222 (.37)	109 (.18)	15 (.43)	6 (.17)
	(.60)	(.15)	-	69 (.12)	200 (.33)	4 (.11)	10 (.29)
Attitude	408	427	+	82 (.20)	96 (.24)	72 (.40)	12 (.07)
	(.40)	(.85)	-	131 (.32)	99 (.24)	17 (.09)	80 (.44)

We first investigated the effects of the manipulated variables on the proportion of Memory-set responses. We expected (and found) the memory manipulation to be reflected in a higher proportion of Memory-set responses for paired target CSs as compared to non-paired foil stimuli: The majority of paired CSs (60%) were judged ‘remembered’ (i.e., received Memory-set responses); only a minority (15%) of nonpaired foils was also falsely ‘remembered’ as having been paired with a US.^9^

Next, we analyzed frequencies of “pleasant”/“unpleasant” responses for paired CSs as well as nonpaired foils. Overall, these judgments were affected by main effects of US Valence (*β̂* = 0.73, 95% CI [0.38,1.07], *z* = 4.12, *p* < .001) and CS Valence (*β̂* = 1.95, 95% CI [1.48,2.41], *z* = 8.26, *p* < .001). Importantly, the US Valence effect was again modulated by Response Set (i.e., it differed between Memory and Attitude-set responses, *β̂* = 0.91, 95% CI [0.59,1.22], *z* = 5.56, *p* < .001). In addition, Response Set also interacted with CS Valence, with smaller CS Valence effects on Memory than Attitude judgments, *β̂* = − 0.65, 95% CI [− 1.05,− 0.24], *z* = − 3.11, *p* = .002.^10^ To break down these findings, we report separate analyses by item type (CSs vs. foils) and Response Set (Memory vs. Attitude), which jointly define the four quadrants of Table 2.

We focused first on paired CSs, for which “pleasant”/“unpleasant” responses CSs showed the same pattern of effects as obtained in the overall analyses reported above. Most importantly, the interaction of US Valence with Response Set (Memory vs. Attitude) was robustly significant, *β̂* = 0.93, 95% CI [0.61,1.26], *z* = 5.63, *p* < .001, suggesting that US Valence effect on judgments differed between Memory-set and Attitude-set responses. For paired stimuli, Memory-set judgments (top left quadrant) quite accurately reflected actual US valence (albeit with a somewhat a lower accuracy than in Experiment 1): When participants experienced remembering US valence for a paired stimulus, they were accurate in 70% of cases. The strongest effect on Memory-set judgments was exerted by US Valence, *β̂* = 1.66, 95% CI [1.22,2.10], *z* = 7.42, *p* < .001 (i.e., “pleasant” responses were more likely for CSs paired with pleasant USs). Additional (smaller) effects were obtained for CS Valence, *β̂* = 1.08, 95% CI [0.46,1.69], *z* = 3.44, *p* = .001 (with pleasant CSs more likely judged “pleasant”, reflecting attitude-consistent guessing), and Material, (with face CSs less likely to be judged “pleasant”, perhaps reflecting the finding that pleasant face CSs were less likely classified using the ‘Memory-set’ responses).^11^

#### Is EC observed without feelings of remembering US valence?

Next, Attitude-set judgments for paired CSs were analyzed, with a particular focus on whether or not they are influenced by US valence in a similar manner as were Memory-set judgments. However, as reflected in the bottom-left quadrant of Table 2, those CSs were not evaluated in accordance with US valence. In a logistic regression, only a CS Valence effect on these judgments was found, *β̂* = 3.82, 95% CI [1.12,6.51], *z* = 2.78, *p* = .005 (with initially pleasant CSs more likely judged pleasant). Importantly, replicating the absence of an EC effect in the absence of feelings of remembering US valence, US Valence did not affect these judgments, *β̂* = 0.06, 95% CI [− 0.81,0.93], *z* = 0.13, *p* = .897.

As in Experiment 1, we additionally computed a *BF* on the proportions of responses in line with US Valence for paired neutral CSs that received an Attitude-set judgment. Going beyond failing to find an EC effect for Attitude-set responses, the Bayesian analysis yielded evidence against above-chance responses in line with US valence (*M =* 0.41; *SD =* 0.20). This supports the absence of (even small) ECeffects in the absence of experiences of remembering, *BF*_*H1*:*N*(0,0.15)_ = 0.07 (Robustness Region: 0.05, 1).

#### Is there evidence for an affect-as-information bias?

Pleasantness judgments of nonpaired foils (i.e., stimuli not presented together with USs) varied only as a function of pre-experimental valence, *β̂* = 2.68, 95% CI [1.59,3.76], *z* = 4.85, *p* < .001, and this effect was not modulated by Response set, *β̂* = − 0.51, 95% CI [− 1.31,0.29], *z* = − 1.26, *p* = .209. For those (relatively few) foils for which participants falsely ‘remembered’ that a US had been paired with them (i.e., used the Memory response set; top right quadrant), they reported a US valence on the novel task that tended to be consistent with pre-experimental attitudes: A stimulus falsely judged as having been paired with a US was evaluated in accordance with pre-existing attitudes in 72% of cases. However, note that in a logistic regression of this relatively small subset of responses, the effect of pre-existing valence (while descriptively consistent with the overall analysis) was not significant, *β̂* = 1.86, 95% CI [− 0.55,4.27], *z* = 1.51, *p* = .131.

Focusing on the bottom right quadrant (i.e., Attitude-set judgments for nonpaired stimuli), it is apparent that judgments reflected pre-experimental attitudes, *β̂* = 3.36, 95% CI [2.05,4.68], *z* = 5.03, *p* < .001: Participants consistently reported the attitude corresponding to their pre-study ratings in 84% of cases. Besides this strong effect of CS Valence, the logistic regression analysis did not yield other significant effects.

In sum, responses on the TBS task again appropriately reflected the memory and attitude manipulations. We replicated the absence of EC effects when participants reported they had no feeling of remembering the valence of the US paired with a given CS (i.e., the effect of US Valence was limited to Memory-set responses). We also replicated the attitude-as-information bias (i.e., the effect of CS Valence on Memory-set responses), which was now obtained not only for nonpaired foils but also for paired CSs.

### Discussion

Confirming the manipulation’s effectiveness, Experiment 1, results again confirmed that the TBS task adequately reflects memory and attitude manipulations (i.e., Memory-set responses separated paired from non-paired stimuli and selectively reflected US valence effects; Attitude-set responses reflected pre-learning evaluative ratings).

Similar to Experiment 1, Experiment 1, here we orthogonally manipulated US valence and CS valence and found an effect of attitudes-as-information (i.e., CS valence) not only for nonpaired foils but also for paired CSs.

In other words, participants were able, to different degrees, to selectively use the two sources (CS and US) of valence in their judgments: On the one hand, US valence had an influence only on Memory-set but not Attitude-set judgments. On the other hand, the effects of CS valence (although less selective) were stronger on Attitude-set judgments (where they reflected valid responding in adherence with task instructions) than on Memory-set judgments (where they reflected an attitude-as-information bias).

By presenting participants with valent as well as neutral items, and with paired as well as non-paired items, Experiment 2 was able to separate effects of pairing from those of (pre-existing) evaluations. The use of this relatively complex design was required by our validation effort. However, it departed from simpler designs typically used in EC studies, which generally present only neutral CSs paired with either pleasant or unpleasant USs. For this reason, one may argue (1) that our primary validation goal is successful, but (2) that TBS conclusions regarding memory-EC relations were affected by—or even originated from—this unusual level of procedural complexity. To examine this possibility, we ran a third experiment that made use of the simpler design typically used in EC studies.

## Experiment 3

Experiment 3 examines whether EC is found when US valence is not remembered, (1) with higher statistical power, and (2) by using a simplified design (i.e., a design without nonpaired and valent stimuli that is more typical for EC studies). In doing so, this experiment provides what may be considered a more liberal (or even fairer) test for EC effects without remembering US valence. This was done in a first condition.

In a second condition, we went beyond this first objective and replicated a study by Waroquier et al. (2020), who reported EC effects when participants indicated that they did not have conscious knowledge about US valence. In their study, after the learning phase, participants were presented with a CS and asked to report the valence of the associated US. They were then asked to indicate the basis of their valence-memory response, that is, to attribute their response to one of several different mental states: *memory* (i.e., when they felt they remembered the pairing), *gut-feeling* (i.e., when they had an intuition or feeling of familiarity about the US’s valence), or *random guessing*. In their Experiment 1, Waroquier et al. found EC effects not only for *memory* but also for *gut-feeling* attributions; in their Experiment 2, EC was found for *memory* and *random-guessing*, but not for *gut-feeling* attributions. These results contrast with the TBS finding of no EC in the absence of feelings of remembering US valence, suggesting that a more fine-grained distinction of different mental states (i.e., three rather than two), and/or a more sensitive measurement (i.e., continuous rating scale rather than binary choice) may be more suited for detecting EC without feelings of remembering.

To begin exploring possible reasons for such a discrepancy, we also included a third condition in which we adapted the TBS approach of conditionally assessing either memory or attitudes as a function of remembering US valence. Specifically, we offered three (rather than two) attributions; and we replaced the binary attitude judgments by continuous evaluative rating scales (i.e., a three-attribution, continuous evaluation task, hereafter 3ACE). Participants’ task was to assess their feelings of remembering US valence; if they remembered it, they were to report it by using binary pleasant/unpleasant buttons as in the TBS; if they had some gut feeling or intuition, they were asked to evaluate the CS on a rating scale; likewise, if they felt they were guessing randomly, they were asked to evaluate the CS on yet another rating scale. With these modifications, the data acquisition rationale was similar to Waroquier et al. (2020), but with a higher degree of adherence to the immediacy criterion (i.e., the data were obtained from a single response at a single point in time, rather than separated across trials or even separate blocks).

In sum, we implemented a between-subjects manipulation of valence-memory task type with three levels: Participants completed either the TBS task, Waroquier et al.’s (2020) valence-memory attribution (i.e., VMA) task, or the 3ACE task. We also counterbalanced the order of post-experimental evaluative ratings and the valence-memory task (which had no effect on the results).^12^

### Method

The study was preregistered at https://osf.io/pducs. Material, data, and analysis scripts are available at https://osf.io/ygcz4.

#### Participants and design

Participants were randomly assigned to a 3 (valence-memory task: TBS, VMA, 3ACE) x 2 (order: evaluative ratings first vs. valence-memory task first) between-subjects design. They were recruited from the lab database (i.e., University of Cologne student population) and were invited to participate either in the lab or via Internet. As compensation, course credit or an amount of EUR 8 was offered. We recruited participants week by week until there were at least 180 participants who had passed the instruction-understanding, seriousness, and attention checks (see also below); this resulted in a final sample size of 185 participants.

#### Material

We used the same face images as Waroquier et al. (2020), but replaced the IAPS images (which cannot be used in online studies for license reasons) by comparable images from the OASIS database. Participants pre-rated 60 images, and the 40 most neutral ones were used as CSs in all subsequent study phases. Half of them were assigned to be paired with positive USs, the other half with negative USs; each CS was paired with a single US. An additional set of three US images of the same valence were assigned to each CS to be used as foils in the test of US identity memory.

#### Procedure

The procedure was similar to the previous studies (with procedural details closely following those from Waroquier et al., 2020). After collecting pre-study ratings (on a scale from -200 to + 200), the learning phase began, presenting 240 trials in which a CS and a US were shown simultaneously and side by side on the screen for 1500 ms (with a 100-ms ISI). Participants were instructed to carefully watch all the stimuli. As an attention check, they were also asked to press the space bar as quickly as possible whenever a fixation cross was presented in the center of the screen. After the study phase, half of participants first gave post-study ratings of all 40 CSs and then engaged in the valence-memory task (i.e., TBS, VMA, or 3ACE); the other half completed the tasks in the reverse order (i.e., valence-memory task first, ratings second). After the valence-memory task instructions were given, participants’ understanding was probed as in Experiment 2. The respective valence-memory task commenced after instruction checks were completed; afterwards, half of participants completed the post-learning evaluative ratings for the 40 CSs. For exploratory purposes, we subsequently presented participants with another valence-memory task (VMA or TBS), followed by a US-identity memory task (see above). Finally, participants were asked whether they paid attention during the learning phase, whether they thought their data should be analyzed for scientific purposes (or were subpar for any reason), and about the setting in which they completed the study (in the lab; alone in a quiet setting; a quiet setting with other people; with other people and occasional interruptions; a lively environment with frequent interruptions).

### Results

We first report results of a manipulation check (i.e., whether there was a robust EC effect). Next, we turn to the results of the TBS task, focusing on whether the absence of EC without feelings of remembering US valence is replicated when using a simplified design. Subsequently, we report EC effects for the different attributions in the VMA and 3ACE tasks. In Appendix A, we report additional results on the proportions of Memory/Attitude responses (in the TBS task), attributions (in the VMA and 3ACE tasks), and on valence memory accuracy in each response set for each task.

#### Evaluative conditioning

**Fig. 7:**
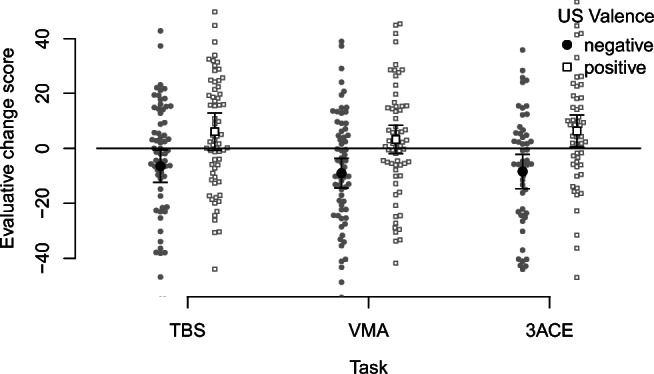
Pre-post evaluative change as a function of US Valence and Task condition. *Dots* are the individual observations, and *error bars* are the 95% confidence intervals. *Note:* TBS = Two-buttons-sets procedure; VMA = Valence Memory Attribution task; 3ACE = Three-attribution, continuous evaluative ratings task

As a manipulation check, we first tested whether there was an EC effect overall, based on the final total sample (*N =* 185). An ANOVA of the pre-post evaluative change scores tested whether they were influenced by US Valence (see Fig. 7), and whether this influence was modulated by task or order condition.

Overall, we found only an effect of US Valence (i.e., an EC effect): Pre-post evaluative change scores were higher for CSs paired with positive than negative USs, *F*(1,179) = 38.34, *p* < .001, *η̂G*2 = .075, 90% CI [.025,.144] (all other effects were not significant).

#### Two-buttons-sets procedure

We conducted analyses with data from participants in the TBS task condition (*n*= 67). As in Experiments 1 and 2, the responses on the TBS task are first given in a tabulated form (Table 3).

**Table 3:** Experiment 3, TBS response frequencies (proportions)

Set	CS	Response	CS +	CS−
Memory	1181	+	475 (.4)	158 (.13)
	(.44)	-	117 (.1)	431 (.36)
Attitude	1499	+	339 (.23)	341 (.23)
	(.56)	-	409 (.27)	410 (.27)

First, we tested accuracy of Memory set responses (i.e., the accuracy of participants’ US Valence reports when they felt they remembered US Valence). The first two rows of the table (“Memory” responses; see columns *CS. +* and *CS.−* for responses on CS paired with positive and negative US, respectively) show that participants accurately distinguished between CS that were paired with positive and negative US when they made a Memory response, *χ*^2^(1) = 336.52, *p* < .001 (see also below).

To analyze participants’ responses in the TBS task, we conducted a mixed-effect logistic regression (random intercepts and slopes for US Valence) with US Valence and Response buttons set as predictors. The interaction between US Valence and Response buttons set was significant, *β̂* = 0.68, 95% CI [0.58,0.78], *z* = 13.39, *p* < .001. We conducted separate analyses at each Response buttons set level to simplify interpretation of the findings. When participants used the Memory response buttons set, the US Valence effect was significant, *β̂* = − 1.72, 95% CI [− 2.11,− 1.33], *z* = − 8.64, *p* < .001, indicating above-chance valence memory accuracy.

In contrast, in the Attitude response buttons set, no significant effect of US Valence was found, *β̂* = − 0.01, 95% CI [− 0.12,0.10], *z* = − 0.18, *p* = .856. This replicates our previous findings of the absence of an EC effect when participants reported having no US valence memory. Importantly, we also found evidence for the absence of an EC effect in a one-sample *t* test against chance level (i.e., .5) on the proportions of TBS responses in line with US valence (*M* = .50; *SD* = 0.09), which were not significantly different from the chance level, *t*(63) = − 0.25, *p* = .807, *d* = − 0.03. As in Experiment 2, a Bayesian analysis yielded evidence for no EC effect in the absence of feelings of remembering US valence, *BF*_*H1*:*N*(0,0.15)_ = 0.061 (Robustness Region: 0.05, 1) (note that this conclusion again holds even for small effects).

#### Additional results

Here, we report the results of our attempt to replicate the EC-without-conscious-knowledge findings by Waroquier et al. (2020), who assessed participants’ attributions of their valence-memory decisions (i.e., subjective assessments of their knowledge) and found EC effects when participants indicated having only intuitions about US valence or when participants reported randomly guessing. We also report initial results from a new version of the procedure (i.e., 3ACE) that serves to illustrate how the TBS approach could be further developed by capturing three (rather than two) retrieval experiences, and by assessing evaluations on a continuous rating scale (rather than as a binary choice).

#### Valence Memory Attribution (VMA) task

We tested whether we replicate Waroquier et al.’s (2020) findings. In particular, we were interested in whether we find an EC effect when participants reported no memory (random guessing). Because the VMA task only assesses valence memory (not evaluations), EC effects were computed as pre-post differences in evaluative ratings as in Waroquier et al. (2020).

We found an EC effect when participants made a memory attribution, *F*(1,65) = 17.65, *p* < .001, *η̂G*2 = .153, 90% CI [.043,.289]. When participants made an intuition attribution, the EC effect was not significant, *F*(1,64) = 2.01, *p* = .161, *η̂G*2 = .011, 90% CI [.000,.087], and *BF* was inconclusive, *BF*_*H1*:*N*(0,23.5)_ = 0.86 (Robustness Region: 0.05, 63.3).^13^

Critically and contrary to Waroquier et al. (2020), we found no EC effect when participants made a random guessing attribution, *F*(1,61) = 1.31, *p* = .257, *η̂G*2 = .007, 90% CI [.000,.078], and *BF* yielded evidence against an EC effect, *BF*_*H1*:*N*(0,23.5)_ = 0.089 (Robustness Region: 5.75, > max).^14^

#### Three-attribution, continuous evaluation (3ACE) task

In the 3ACE data, we found an EC effect when participants made a memory attribution, *F*(1,50) = 17.70, *p* < .001, *η̂G*2 = .183, 90% CI [.048,.339]. When participants made a feeling/intuition attribution, the EC effect was also significant, *F*(1,48) = 8.79, *p* = .005, *η̂G*2 = .054, 90% CI [.000,.186]. We found no EC effect when participants made a random-guessing attribution, *F*(1,46) = 2.30, *p* = .136, *η̂G*2 = .012, 90% CI [.000,.110], and the *BF* yielded evidence against an EC effect, *BF*_*H1*:*N*(0,23.5)_ = 0.11 (Robustness Region: 7.35, > max).^15^

### Discussion

Across tasks, robust EC effects were found for Memory-set responses and memory attributions, and EC effects were clearly absent for Attitude-set responses and random-guessing attributions. This pattern of results is in line with those of Experiments 1 and 2, which also found an EC effect when participants felt they remembered US valence, but not when participants felt they did not remember. These results support the robustness of the conclusions derived from the TBS task. In particular, the same results are obtained regardless of whether nonpaired and valent stimuli are included in the design or not. Note that, with nonpaired and valent items included, the TBS is a much more powerful tool, especially for investigating the attitude-as-information bias and possibly other heuristics.

Regarding gut-feeling (or intuition) attributions, results diverged between Waroquier et al. (2020)’s Valence Memory Attribution (VMA) task and the 3ACE task we introduced in Experiment 3: While the evidence for or against an EC effect with gut-feeling attributions was inconclusive in the VMA, the 3ACE yielded positive evidence for such a gut-feeling EC effect. The present evidence for the absence of EC in the absence of remembering US valence contrasts with the finding of EC for “random guessing” cases in Waroquier et al.’s (2020) Experiment 1 and 2. Given that our study is a close replication of the studies by Waroquier, we can only speculate about the reasons for the discrepancy (i.e., there were differences in participant populations, US stimuli, language of instructions); more research is needed to try to replicate their EC effect for random-guessing attributions and to explain why the current Experiment 2 failed to do so. With regard to the “intuition” attributions, our findings are more in line with those by Waroquier et al., who also found inconclusive evidence for these cases (i.e., there was evidence for feeling-based EC in Exp.1, but evidence was inconclusive in the immediate test of Exp.2, and there was evidence for the absence of EC on the delayed test). Interestingly, we found an EC effect for “intuition” attributions in the 3ACE task in which attributions and evaluative ratings were collected in a single task (hence better respecting the immediacy criterion than the VMA task). To identify the factors underlying a potential “intuitive” or “gut-feeling” EC effect, future research should aim at better understanding the subjective state of gut-feeling attributions, and its relation to similar subjective memory reports (such as “know” judgments that are often associated with familiarity-based or semantic memory retrieval).

## General discussion

The present research introduced a new conditional judgment procedure - the TBS task - for advancing the study of (potential) EC effects without feelings of remembering US valence. In the empirical section, we validated this new task, and we additionally used it to deliver preliminary results on attitude-as-information biases typically found in EC studies interested in memory, as well as on the question of EC effects in the absence of feelings of remembering US valence. In this General discussion, we first discuss the main findings observed here in light of these main objectives. Next, we will address the limitations of the novel approach, and explain how it can be further improved and used to advance research on attitudes and beyond.

### Validity of TBS task

Our primary aim was to test the validity of the TBS task. In two experiments, we influenced memory by pairing some stimuli with USs, while other stimuli were never paired with USs; and we influenced attitudes by using stimuli pre-rated as pleasant or unpleasant. The manipulations of pairing memory were reflected in participants’ metacognitive judgments (i.e., their use of the Memory-set vs. Attitude-set), as well as their valence-memory reports when they used the Memory set. The manipulations of attitudes were reflected in participants’ pleasantness judgments when they used the Attitude set. Experiment 3 further supported the robustness of conclusions derived from the TBS: similar conclusions about EC effects without feelings of remembering US valence were obtained when excluding (the otherwise informative) nonpaired and valent stimuli from the design. These results confirm a successful validation of the TBS task (comparable to that of the process-dissociation procedure by Hütter et al., 2012).

### Evaluative conditioning and feelings of remembering

A more secondary aim was to use the TBS to provide preliminary information about whether EC requires feelings of remembering US valence. Using the TBS task, we collected CS-level data on evaluations in the absence of feelings of remembering US valence. While CS evaluations in the absence of feelings of remembering US valence reflected preexisting attitudes (attesting to their validity), they were however consistently unaffected by US valence. To the contrary, we found evidence for the *absence* of EC effects when participants felt they did not remember US valence.

This result is largely in line with results from the process-dissociation task as summarized by a recent meta-analysis of PD studies (Mierop et al., 2018). Across 12 PD studies, this analysis yielded an overall mean estimate of *A*=.07, 95% CI [.04, .11]. First, this is a relatively smalleffect; it corresponds to approximately 1 out of the 24 CSs typically presented to a given participant being governed by the “EC in the absence of memory” branch of the MPT model.^16^ Second, the meta-analysis also revealed substantial heterogeneity (*I*^2^ = 56*%*), reflecting the fact that the confidence interval of *A* excluded zero in five out of 12 studies, whereas it included zero in the remaining seven studies. In sum, while PD studies yield some meta-analytic evidence for the persistence of EC effects in the absence of US valence memory, the effect is small and contingent on unknown enabling conditions. Given sample sizes comparable to (and, jointly, considerably larger than) previous studies, the present null results are unlikely to reflect lack of power. Indeed, Bayes factors indicate evidence for the absence of even small effects. It remains possible, of course, that EC without remembering US valence will be found in other studies that realize (as yet elusive) critical enabling conditions.

The finding of no EC without feelings of remembering US valence is fully in line with previous conclusions obtained with per-item procedures that also found no EC when participants indicated not remembering the valence of the paired US (e.g., Stahl et al., 2009). It is however at odds with a recent per-item result by Waroquier et al. (2020) showing EC even when participants indicated having randomly guessed whether the US’s valence was positive or negative. In the present Experiment 3, we failed to replicate the latter finding. However, assuming that the findings by Waroquier et al. can be replicated in future studies, there are three important differences between the TBS and their approach that could account for the inconsistent result patterns. First, while the present TBS task allowed for only two different metacognitive states (i.e., participants could report either remembering or not-remembering), the task used by Waroquier et al. required a finer distinction of three or four metacognitive states (i.e., memory, intuition, familiarity, random guessing). On the one hand, such a fine-grained distinction may put too high a demand on participants, especially if instructions fail to clearly explain the different states (for a discussion in the context of the related Remember/Know procedure see Migo et al., 2012). On the other hand, a finer-grained distinction may be required to validly tap relevant metacognitive states (e.g., structural vs. judgment knowledge, Dienes et al.,, 1995, Dienes & Scott, 2005). Second, in Waroquier et al.’s studies, the EC effect was assessed using a continuous rating scale, whereas a binary choice was used in the present TBS studies; the former may capture more subtle effects and could therefore be more sensitive than the latter method. As a final difference, while in the TBS task participants reported their metacognitive state simultaneously with the pleasant/unpleasant judgment, valence-memory and metacognitive attribution were assessed in separate trials (Exp. 1) or even in separate blocks (Exp. 2) in the studies by Waroquier et al., (2020). With a delay between judgments, their metacognitive measure met the immediacy criterion less well than the present TBS task. One may, therefore, speculate that their results reflect the addition of noise to the evaluation-memory relation, which could lead to an artificial “EC without feelings of remembering US valence” pattern. Future task developments could move towards a task such as the 3ACE used in Experiment 2 that respects the immediacy criterion, allows for more than two attributions (e.g., that distinguishes not only memory and no-memory states but also allows for intuition attributions), and uses a more sensitive response format (i.e., a continuous rating scale rather than a binary choice).

The finding of an EC effect for gut-feeling attributions in the 3ACE points to the possibility of EC in the absence of feelings of remembering US valence. It remains open whether these findings reflect some participants’ conservative decision criteria (i.e., a tendency to respond “intuition” for somewhat weaker remember experiences) or a distinct process altogether. Clearly, however, this finding (if robust) will be important both for research into the processes underlying evaluative memories as well as for applied research targeting strategies for controlling persuasive influences.

Whether, when, and through which processes EC effects can be found in the absence of feelings of remembering US valence bears important theoretical implications. The evaluative conditioning literature distinguishes between Stimulus-Stimulus and Stimulus-Response evaluative learning models. Stimulus-Stimulus models assume that CS–US links are encoded in memory and that their retrieval influences participants’ judgments at the decision stage (e.g., Aust et al., 2019; Stahl & Aust, 2018). For these models, EC effects are contingent upon a successful retrieval of the CS–US pairings from memory. Alternatively, Stimulus-Response models assume that the affective responses elicited by the US (rather than the US as such) are linked to the CS in long-term memory (for a recent discussion, see Corneille & Stahl, 2019; see also Jones, Fazio & Olson, 2009; Sweldens, Van Osselaer & Janiszewski, 2010). For these models, CS exposure directly cues the learned affective response. Hence, neither the encoding nor the retrieval of a CS–US pairing is required to observe EC effects.

Stimulus-response learning is thought to operate via a “direct transfer” of the US valence to the CS (e.g., Hütter & Sweldens, 2018; Sweldens et al.,, 2010) or via implicit misattribution. The latter is thought to occur when, at the learning stage, participants mistakenly attribute their affective response to the CS instead of attributing it to the US (e.g., Jones et al.,, 2009). In turn, implicit misattribution processes are expected to operate under specific conditions that are conducive to confusability about the source of one’s affective reaction (Hütter & Sweldens, 2013; Jones et al., 2009). These conditions were not met in the learning paradigms used for the present research. Recent evaluative conditioning research, however, casts doubts on the existence of stimulus-response learning in more conducive conditions. In particular, evidence for unaware EC effects in the original paradigm (Olson and Fazio, 2001), failed to be supported in more recent studies that relied on this paradigm (Moran, 2020; Kurdi et al., 2022). Another piece of evidence for stimulus-response learning, relating to source confusability, was also compromised in recent studies. Specifically, implicit misattribution assumes stronger EC effects when US valence is mild rather than high. This is because a very valent US is unlikely to elicit confusion about the source of one’s affective reaction. Recent research failed to find evidence consistent with this rationale (Mierop et al., 2018). Finally, that a learning effect on the memory-independent parameter has been found in instruction-based procedures (Hütter & De Houwer, 2017) further challenges an associative interpretation of the findings (for related demonstrations, see also Béna et al.,^2022^; Corneille et al.,, 2019).

### Affect-as-information bias

When participants lack US valence memory, they may use their evaluations to inform their memory judgments; in that case, a potential “EC without memory” effect is masked. To circumvent this problem, per-item tasks have asked participants to indicate when they do not remember US valence (e.g., by responding “I don’t remember”), and the process-dissociation and TBS tasks have asked them to report their attitudes (instead of US valence memory) whenever they feel they do not remember. However, more recently it was found that attitude-as-information bias may also affect metacognitive “remember” states (Bar-Anan & Amzaleg-David, 2014; Bar-Anan et al., 2010), and here we assessed whether this is the case also for US valence memory judgments in the TBS task. In support of this possibility, Memory-set judgments of nonpaired foil stimuli were affected by participants’ attitudes towards these stimuli.

Thus, attitude-as-information bias was obtained for cases for which participants had a certain amount of confidence in their memory, as they indicated remembering US valence (i.e., used Memory-set responses on the TBS task), and despite the option to refrain from reporting US valence memory (i.e., they could have reported an Attitude-set or “don’t remember” response). These observations are at odds with the suggestion that the attitude-as-information bias stems from a heuristic used under uncertainty that is linked to forced responding; instead, they are consistent with findings reported by Bar-Anan et al., (2010, 2014) suggesting that cases of attitude-consistent responding may be experienced, subjectively, as true remembering. Additional research is needed to address this intriguing phenomenon, as well as its consequences for the controllability of evaluative-conditioning influences on attitudes and behavior.

### Limitations and open questions

The TBS is limited in that it (1) addresses only deliberate evaluations, (2) distinguishes only two metacognitive states, and (3) addresses only a very specific issue. Here we discuss these limitations and sketch possible ways to address them in future developments. Before we do so, it is important to highlight that both the TBS and Hütter et al.’s (2012) PD procedure taps awareness of knowing rather than intentional control.

#### Intentional control versus awareness of knowing

Researchers familiar with the literature on process dissociation, which capitalizes on the notion of (un)intentional control, may be hesitant to conclude that the TBS task is indeed an improvement upon the PD approach. Tapping the ability to intentionally control information is critical in many traditional process-dissociation tasks, which typically aim at dissociating two distinct contributions to behavior, only one of which is under intentional control. The inclusion-exclusion instruction manipulates how the controllable process is mapped onto the set of responses, and it is assumed that the uncontrollable process affects responses in the same way in both instruction conditions. In other words, participants in process-dissociation tasks can only reverse the response assignment in the exclusion condition for intentionally controllable information; the response pattern remains unaffected by the instruction manipulation insofar as there are also uncontrollable contributions.

In the present research, instead of intentional control, awareness of knowing is of central interest. Per-item measures of valence memory require judgment knowledge (i.e., whether a CS had been paired with positive or negative USs), as well as their awareness of knowing (i.e., participants are asked to respond “I don’t remember” if they are unaware of any knowledge). The TBS similarly taps these two aspects of awareness, albeit in a slightly different way (i.e., it asks participants to report their attitude if they are not aware of having any knowledge). For both per-item and TBS tasks, we assume that knowledge about one’s subjective knowledge state (i.e., being aware of having knowledge or not) can be used intentionally (i.e., participants intentionally select whether to report their memory or their attitude).

Importantly, controlling the use of a piece of information or knowledge is not the same as being aware of knowing it (i.e., control may be possible for unconscious knowledge, that is, for which awareness of knowing is lacking; it is also conceivable, at least, that awareness of knowledge may be present in some situations while that knowledge uncontrollably affects a specific task). Hence, if the TBS and the PD approach as applied to assessing EC without memory by Hütter et al., (2012) were to capture distinct features of consciousness, this might explain different patterns of results (and would prohibit presenting the TBS task as an improvement of Hütter et al.’s PD approach).

However, while it may generally be possible to control the use of unconscious information strategically (i.e., in the absence of awareness of knowing), this does not characterize the PD task as it is applied to EC by Hütter et al., (2012). Participants in their PD task also require awareness of knowing as well as judgment knowledge in order to follow instructions: To be able to select the relevant part of the instruction (i.e., to report memories or attitudes), participants have to decide whether, subjectively, they have knowledge or not (i.e., they need awareness of their knowledge); to be able to report their attitude (or memory), they require judgment knowledge (i.e., regarding whether a CS had been paired with positive or negative USs). Importantly, this knowledge must be under their intentional control, so that they can follow the respective inclusion or exclusion instruction (i.e., report veridical versus reversed attitudes or memories). This is highlighted by the fact that Hütter et al. proposed two exchangeable versions of their task, one in which instructions require reversal of memory judgments, and another in which instructions require reversal of attitude judgments (although we have discussed here only the more widely used attitude-exclusion variant). In this sense, the PD approach to assessing EC without memory is not a traditional PD paradigm that capitalizes on (un)controllability of (in this case) attitudinal information — to the contrary, it assumes and requires attitudinal information to be controlled. It hinges on the subjective assessment of having (versus not having) conscious knowledge about CS–US pairings; it is this subjective awareness of (not) knowing that determines whether a response is reversed or not.^17^

#### Deliberate memory retrieval and evaluations

The TBS relies on deliberate evaluations; results and conclusions may differ when so-called “implicit” attitude measures are used; and future research is needed to relate the results of direct and indirect measurement approaches. It is unclear, however, on which basis “explicit” and “implicit” attitude measures should be distinguished from each other. At present, the distinction reflects social convention rather than being grounded in a compelling empirical or theoretical rationale (for a thorough discussion, see Corneille & Hütter, 2020). If “implicit” is understood as “automatic”, then the TBS clearly allowed for the influence of unconscious (memory) processes. This is also true for deliberate evaluations used in Process Dissociation studies. We note that the TBS could be adapted for addressing other features of automaticity, such as efficient responding, for example by requesting speeded evaluations.

Relatedly, the TBS explicitly asks participants whether they felt they remembered the CS–US pairings; this instruction should induce them to try to retrieve their memory for the pairings. What we found is that EC is only observed when participants felt they remembered US valence. However, it is possible that participants would show EC effects without such feelings of remembering, if they were not explicitly asked to retrieve the valence of the USs paired with the CSs. In other words, whereas EC may require CS–US pairing memory, it may not in all circumstances require feelings of remembering of those memories at the time of CS evaluations. This theoretical possibility could be tested, for instance, with the help of indirect measures of memory in future studies.

Another question is whether different conclusions may be reached when using physiological measures. For instance, studies have claimed successful subliminal conditioning for salivating response but not for evaluations (Passarelli et al., 2022). Although recent research suggests the involvement of convergent evaluative learning processes for behavioral (direct or indirect) and physiological measures (Corneille and Mertens, 2020), one should be open to the possibility that different learning or decision making processes are operating when specific physiological responses, or specific populations, are under consideration. Likewise, one may speculate that behavioral measures capitalizing on preference rather than absolute evaluative judgments (Amd & Passarelli, 2020) may be more sensitive to memory-independent processes (but see Heycke et al.,, 2017). Finally, one should be careful not generalizing the conclusions reached here in the context of evaluative conditioning to other forms of learning from pairings, such as reward learning (Leganes-Fonteneau et al., 2021).

#### Coarse metacognitive distinction

The TBS distinguishes between only two different metacognitive states (i.e., having feelings of remembering or not); it does not allow participants to report other metacognitive states. In particular, several important dissociations have been made in previous research, for instance between remembering versus knowing (Wixted & Mickes, 2010), or structural versus judgment knowledge (Dienes & Scott, 2005). The TBS task is however easily extended to allow for more fine-grained metacognitive distinctions. For instance, an adapted version (such as the 3ACE task piloted in Experiment 3) could offer three attribution options (one each for feelings of remembering, gut feeling, and guessing) to more conclusively assess whether the results obtained by Waroquier et al., (2020) reflect an artifact due to lack of immediacy, or whether their more fine-grained metacognitive distinctions were better able to isolate the relevant cases than the present TBS task. The EC effect for gut-feeling/intuition attributions found on the 3ACE (which respects the immediacy criterion better than Waroquier et al.’s task) points to the latter. More research is needed to understand better when and why EC effects could be obtained without feelings of remembering.

#### Going beyond the EC-without-feelings-of-remembering issue

The TBS task was used here for addressing one very specific research question in attitude research, but the task (or the general conditional-judgment approach) may be adapted for addressing other, similarly structured questions. For instance, there is a related issue in research on truth judgments, which depend on participants’ memory for the statement in question: It is typically found that truth judgments are higher for old, repeated statements than for new ones (Dechêne et al., 2010). The TBS task could be adapted to assess perceived truth instead of pleasantness, and separate truth judgments in the presence versus absence of feelings of remembering. Previous research has suggested that it is not the factual repetition that increases truth judgments, but that instead a perceived repetition (i.e., a false alarm) is sufficient to increase truth judgments (Bacon 1979; see also Mattavelli et al.,, 2022). In addition to providing a simple assessment of metamemory status, a parallel application of the TBS task to truth judgments could also help investigate whether similar judgment biases affect both types of ratings (e.g., whether statements perceived as more true are also more likely to be judged ‘remembered’). Likewise, mere-exposure effects may be revisited using the TBS. In mere-exposure studies, stimuli are preferred when participants have processed them earlier relative to when they are novel (Zajonc, 1968). Many studies have supported the view that this preference may arise independently of a conscious recollection of the old status of the item (Zajonc, 2001), or even be compromised by conscious recollection (e.g., Bornstein & D’Agostino, 1992; Newell & Shanks, 2007; Whittlesea & Price, 2001). Here too, the use of the TBS may be useful for shedding complementary light on these questions. Finally, recent findings in the literature on artificial grammar learning (AGL) have found evidence for EC in the absence of awareness of knowing (Jurchiş, Costea, Dienes, Miclea, & Opre, 2020). In that study, awareness of knowing and EC were assessed using separate tasks; hence, the result is potentially susceptible to a lack of immediacy. It would be interesting to test whether the finding can be replicated in a variant of the TBS task applied to the AGL paradigm.

### Conclusions and outlook

Studying the relation between EC effects and memory for CS–US pairings informs current (evaluative) learning models. It also bears significant practical implications; for instance, regarding the controllability of persuasive influences in consumer behavior. Here, we introduced and validated a novel conditional-judgment approach (the TBS task) for advancing the understanding of how EC effects relate to feelings of remembering US valence. We argued that the novel approach combines the advantages of the previous per-item and process-dissociation approaches while overcoming some of their limitations. Beyond our primary validation goal, we used the TBS to obtain preliminary insights into EC effects without feelings of remembering US valence and into attitude-as-information effects. We found evidence against the former. In addition, we found evidence for attitude-as-information effects when participants felt they remembered US valence, suggesting that the use of this heuristic is not contingent on memory uncertainty. We also illustrated how the conditional-judgment rationale underlying the TBS task may be adapted to address outstanding research questions of contemporary interest.

## Appendix A: Additional results

**Table 4:** Mean proportions (and SD) of Memory and Attitude-set responses and of valence memory accuracy (with tests against the chance level) in each experiment’s Two-buttons-sets procedure

Experiment/Response set	Proportion of responses	Valence memory accuracy	*t* test	Cohen’s *d* (95% CI)	*B**F*_*H**1*_ (Robustness Region)
Experiment 1					
Memory	.65 (0.18)	.81 (0.15)	*t*(41) = 13.11, *p* <.001	2.02 (1.49, 2.55)	4e + 13 (0.05, 2)
Attitude	.35 (0.18)	.53 (0.23)	*t*(41) = 0.94, *p* = .352	0.15 (-0.16, 0.45)	0.58 (0.05, 0.25)
Experiment 2					
Memory	.55 (0.18)	.73 (0.15)	*t*(35) = 9.04, *p* <.001	1.51 (1.02, 1.98)	2.8e + 08 (0.05, 2)
Attitude	.45 (0.18)	.41 (0.2)	*t*(34) = -2.63, *p* = .013	-0.44 (-0.79, -0.09)	0.07 (0.05, 2)
Experiment 3					
Memory	.44 (0.26)	.76 (0.22)	*t*(64) = 9.89, *p* <.001	1.23 (0.9, 1.55)	9.6e + 11 (0.05, 2)
Attitude	.56 (0.26)	.497 (0.09)	*t*(63) = -0.25, *p* = .807	-0.03 (-0.28, 0.21)	0.061 (0.05, 2)

Note. In Experiments 1 and 2, the analyses were performed only on the responses to the paired neutral conditioned stimuli. ‘*t* test’: two-tailed one-sample *t* tests on the proportions of correct valence responses against chance level (.5). ‘*B**F*_*H**1*_’: Bayes factor quantifying the support for the *H1* (modelled as a half-normal distribution with an SD of 15% see also main text). ‘Robustness Region’: range of SDs of the *H1* distribution that support the same conclusion as the reported *BF* (in the analyses, max SD = 2)

**Table 5:** Mean proportions (and SD) of attributions and of valence memory accuracy (with tests against the chance level) in the Valence Memory Attribution (VMA) and Three-attribution, continuous evaluation (3ACE) tasks (Experiment 3)

Task/ Attribution	Proportion of responses	Valence memory accuracy	*t* test	Cohen’s *d* (95% CI)	*B**F*_*H**1*_ (Robustness Region)
VMA					
Memory	.33 (0.16)	.87 (0.17)	*t*(65) = 17.19, *p* <.001	2.12 (1.68, 2.55)	5.5e + 22 (0.05, 2)
Intuition	.39 (0.15)	.55 (0.16)	*t*(65) = 2.59, *p* = .012	0.32 (0.07, 0.56)	6.2 (0.05, 0.3)
Random guessing	.28 (0.18)	.53 (0.18)	*t*(64) = 1.21, *p* = .231	0.15 (-0.1, 0.39)	0.54 (0.05, 0.2)
3ACE					
Memory	.43 (0.21)	.84 (0.15)	*t*(51) = 16.76, *p* <.001	2.32 (1.8, 2.85)	3.1e + 19 (0.05, 2)
Feeling	.25 (0.13)	.54 (0.19)	*t*(50) = 1.60, *p* = .116	0.22 (-0.06, 0.5)	1.1 (0.05, 0.5)
Random guessing	.32 (0.21)	.45 (0.16)	*t*(48) = -2.23, *p* = .030	-0.32 (-0.6, -0.03)	0.05 (0.05, 2)

Note. ‘*t* test’: two-tailed one-sample *t* tests on the proportions of correct valence responses against chance level (.5). ‘*B**F*_*H**1*_’: Bayes factor quantifying the support for the *H**1* (modelled as a half-normal distribution with an SD of 15%; see also main text). ‘Robustness Region’: range of SDs of the *H**1* distribution that support the same conclusion as the reported *BF* (in the analyses, max SD = 2). ‘VMA’: Valence Memory Attribution task; ‘3ACE’: Three-Attribution, Continuous Evaluation task

## Footnotes

1 Both of the TBS’s questions must be answered by a single response on a single trial, thereby eliminating the temporal distance between retrieval operations. However, the two questions may or may not involve only a single unitary memory retrieval operation. In a modeling framework such as MINERVA, it is plausible that a single operation would be sufficient, with the strength of the memory echo informing the Memory-Attitude decision, and the echo’s content informing the pleasant-unpleasant response (for an application to EC, see Aust et al.,, 2019; Stahl & Aust, 2018). Yet, participants may also approach the task differently, using a two-step approach involving multiple retrieval operations. Future empirical and modeling research is needed to address this question.

2 We used R (Version 4.2.2; (R Core Team, 2019)) and the R-packages *afex* (Version 1.2.1; (Singmann et al., 2022)), *BayesFactor* (Version 0.9.12.4.4; (Morey and Rouder, 2018)), *bfrr* (Version 0.0.0.9000; (DeBruine & Dienes, 2022)), *carData* (Version 3.0.5; (Fox et al., 2022)), *coda* (Version 0.19.4; (Plummer et al., 2006)), *dplyr* (Version 1.0.10; (Wickham, 2021a)), *effects* (Ben-Shachar et al., 2020; Fox, 2003; Fox & Hong, 2009; Fox & Weisberg, 2018; Lüdecke, 2018)), *effectsize* (Version 0.8.2; Ben-Shachar et al., 2020), *forcats* (Version 0.5.2; (Wickham et al., 2021b)), *ggeffects* (Version 1.1.5; (Lüdecke, 2018)), *ggplot2* (Version 3.4.0; (Wickham, 2016)), *kableExtra* (Version 1.3.4; (Zhu, 2021)), *lme4* (Version 1.1.31; (Bates et al., 2015)), *lmerTest* (Version 3.1.3; (Kuznetsova et al., 2017)), *Matrix* (Version 1.5.1; (Bates & Maechler, 2021)), *pacman* (Version 0.5.1; (Rinker & Kurkiewicz, 2018)), *papaja* (Version 0.1.1; (Aust & Barth, 2022)), *psych* (Version 2.2.9; (Revelle, 2022)), *purrr* (Version 1.0.1; (Henry & Wickham, 2020)), *readr* (Version 2.1.3; (Wickham et al., 2022b)), *sjPlot* (Lüdecke, 2021a)), *sjstats* (Lüdecke, 2021b)), *stringr* (Version 1.5.0; (Wickham et al., 2019b)), *tibble* (Version 3.1.8; (Müller & Wickham, 2021)), *tidyr* (Version 1.3.0; (Wickham & Girlich, 2022a)), *tidyverse* (Version 1.3.2; (Wickham, 2019a)), and *tinylabels* (Version 0.2.3; (Barth, 2022)) for all our analyses.

3 A logistic regression confirmed that participants reported more Memory responses for the former than the latter (and vice versa), *β̂* = 2.33, 95% CI [1.83, 2.84], *z* = 9.05, *p* < .001. A model with only Pairing predictor performed better than models including the Valence factor and/or the interaction.

4 Waroquier et al. (2020) chose this value based on Stahl et al. (2009) who found valence memory performance roughly 15% above the 50% chance level; we assume here that effects on memory and evaluations should be comparable under a single-process account.

5 As in Experiment 1, we collected data for an additional TBS Exclusion condition and administered additional memory measures at the end of the study; this information is omitted from the Results section; and the data will be reported elsewhere and made available in the OSF repository.

6 The study aimed for *N* = 120 participants; after the final day of data collection a total of *N* = 123 had participated. As in Hütter et al. (2012), but deviating from Exp. 1, participants (*n* = 13) were excluded who failed to correctly implement the instructions even after reading them for the third time. The total sample consisted of *N* = 110 participants (i.e., an additional *n* = 34 completed a TBS-Exclusion condition not reported here).

7 Given that Experiment 2 was run in a different lab (at the University of Cologne) and implemented in a different programming environment (OpenSesame) than Experiment 1, there may be additional differences between procedures that are not listed here because they have escaped our attention.

8 It could be argued that unfamiliar toy figures are more neutral than human faces, and so the larger EC effect for the toys could be taken to support that notion, albeit applied to classes of materials varied between participants, not variations between CSs within participants.

9 In addition to confirming the effect of the memory manipulation, a logistic regression analysis (which included random intercepts as well as random slopes for US and CS Valence) of the choice of Response Set (Memory vs. Attitude), with CS Valence, US Valence, and Material as predictors, indicated effects of all three experimental factors. We conducted separate analyses of paired CSs and nonpaired foils to simplify interpretation of these findings. For paired CSs, effects of Material and CS Valence were found, as well as interactions of Material with both valence factors. Valent CSs were more often judged ‘remembered’ than neutral CSs, *β̂* = − 0.46, 95% CI [− 0.75,− 0.17], *z* = − 3.12, *p* = .002. Faces were more often judged ‘remembered’ than toy figures, *β̂* = − 0.34, 95% CI [− 0.61,− 0.08], *z* = − 2.52, *p* = .012. An additional Material x CS Valence interaction indicated that the ‘Memory-set’ advantage was observed for negative faces but positive toy figures, *β̂* = 0.63, 95% CI [0.29, 0.97], *z* = 3.62, *p* < .001. Also, toy figures paired with negative (vs. positive) USs received ‘Memory-set’ responses somewhat more often, *β̂* = − 0.25, 95% CI [− 0.50,− 0.01], *z* = − 2.07, *p* = .038. Neither Material (*β̂* = 0.14, 95% CI [− 0.44, 0.73], *z* = 0.48, *p* = .631) nor CS Valence (linear: *β̂* = 0.02, 95% CI [− 0.83, 0.86], *z* = 0.04, *p* = .971, quadratic: *β̂* = − 0.10, 95% CI [− 0.73, 0.54], *z* = − 0.30, *p* = .765) affected judgments of nonpaired foils (and the US Valence factor has only a single level for these items).

10 Additional effects included a main effect of Response Set, *β̂* = 0.26, 95% CI [0.05, 0.46], *z* = 2.45, *p* = .014, a Response Set by Material interaction, *β̂* = − 0.49, 95% CI [− 0.70,− 0.29], *z* = − 4.71, *p* < .001.as well as three-way interactions of Response Set with CS Valence and Material, *β̂* = − 0.28, 95% CI [− 0.56, 0.00], *z* = − 1.96, *p* = .050, and with CS Valence and US Valence, *β̂* = 0.85, 95% CI [0.20, 1.50], *z* = 2.56, *p* = .010.

11 In addition, the above three-way interaction between Response Set, Material, and CS Valence was reflected here as a significant Material by CS Valence interaction, *β̂* = − 0.51, 95% CI [− 0.94,− 0.09], *z* = − 2.37, *p* = .018.

12 Furthermore, to explore the relation between memory judgments on the different memory tasks, we also varied task type within-subjects: Participants completed a second valence-memory task (i.e., participants in the TBS and 3ACE condition completed the VMA task, and participants in the VMA condition completed a TBS task). Finally, to explore the accuracy of subjective memory attributions, participants completed a US identity memory task. The results of these tasks are not reported in the present paper (as it primarily focuses on the validation of the TBS task); procedural details and results will be reported elsewhere (see osf.io/ygcz4).

13 We used the same *H1* prior distribution as Waroquier et al. (2020), who expected EC effects of approximately 23.5 liking units on a 400 point scale.

14 As done by Waroquier et al., we grouped intuition and random attributions into a single “no conscious structural knowledge” category. We did not find an EC effect for this category, *F*(1, 65) = 0.37, *p* = .544, *η̂**G*2 = .002, 90% CI [.000,.050], and *BF* yielded evidence against it, *B**F*_*H**1*:*N*(0,23.5)_ = 0.21 (Robustness Region: 14.85, > max).

15 When feeling/intuition and random guessing responses were grouped into a single “no conscious structural knowledge” category, we did not find an EC effect, *F*(1, 51) = 3.23, *p* = .078, *η̂**G*2 = .021, 90% CI [.000,.124], but the *BF* was inconclusive, *B**F*_*H**1*:*N*(0,23.5)_ = 2.1 (Robustness Region: 12.25, 169.95).

16 With estimates of *M* around .5, valence memory was present for about half of the CSs (i.e., 12 out of 24); of the remaining half, an average of 7% of responses was found to be consistent with the “EC-without-memory” pattern (i.e., 7% of 12 CSs amounts to less than 1 CS).

17 This interpretation is in line with Waroquier et al. (2020) who have also pointed to the subjective nature of Hütter et al. (2012) PD task. It is important to note, however, that the PD approach is not fully subjective: The model parameters provided by Hütter et al., (2012) reflect not only the subjective assessment of having memory but also its accuracy, and not only the assessment of having an attitude towards a CS in the absence of memory, but an attitude in the direction of the valence of the US that was paired with that CS.
